# Supplementary material for: Giant ferroelectric polarization in a bilayer graphene heterostructure
Source: Nat Commun. 2022 Oct 21;13:6241. doi: 10.1038/s41467-022-34104-z (PMC9587233; doi:10.1038/s41467-022-34104-z)
Supplement: Supplementary file 1 — Supplementary Information [file 41467_2022_34104_MOESM1_ESM.pdf]

Supplementary Information

Giant ferroelectric polarization in a bilayer graphene heterostructure

Ruirui Niu[1], Zhuoxian Li[1], Xiangyan Han[1], Zhuangzhuang Qu[1], Dongdong Ding[1], Zhiyu Wang[1], Qianling Liu[1], Tianyao Liu[1], Chunrui Han[2,3], Kenji Watanabe[4], Takashi Taniguchi[4], Menghao Wu[5], Qi Ren[6], Xueyun Wang[6], Jiawang Hong[6], Jinhai Mao[7], Zheng Han[8,9], Kaihui Liu[1], Zizhao Gan[1], Jianming Lu[1*]

[1]State Key Laboratory for Mesoscopic Physics, School of Physics, Peking University, Beijing 100871, China

[2]Institute of Microelectronics, Chinese Academy of Sciences, Beijing 100029, China

[3]University of Chinese Academy of Sciences, Beijing 100049, China

[4]National Institute for Materials Science, 1-1 Namiki, Tsukuba 305-0044, Japan.

[5]School of Physics, Huazhong University of Science and Technology, Wuhan, Hubei 430074, China

[6]School of Aerospace Engineering, Beijing Institute of Technology, Beijing 100081, China

[7]School of Physical Sciences, University of Chinese Academy of Sciences, Beijing 100049, China

[8]State Key Laboratory of Quantum Optics and Quantum Optics Devices, Institute of Opto-Electronics, Shanxi University, Taiyuan 030006, China

[9]Collaborative Innovation Center of Extreme Optics, Shanxi University, Taiyuan 030006, China

<u>Table of contents</u>

Supplementary Notes

1. Additional data and analysis of Device 1 (D1)
    1.1 Identification of moiré features
    1.2 The stacking angles
    1.3 Results of State 1
2. Measurement of Device 2 (D2)
    2.1 The stacking angles
    2.2 Electrical measurement
3. More control devices
4. Discussion about the mechanism

Supplementary Tables

Table S1

Supplementary Figures

Fig. S1-17 for Device 1;

Fig. S18-19 for Device 2;

Fig. S20.

Supplementary References

Supplementary Notes

1. Additional data and analysis of Device 1 (D1)

1.1 Identification of moiré features

As the carrier density accommodated by a moiré band is one of the main focuses, we need to firstly verify that the moiré features shown in Fig. 1b indeed result from the full filling of moiré bands rather than a half or quarter filling. Firstly, Brown-Zak oscillations are usually utilized to quantify the area of supercell. As analyzed in Fig. S2, the superlattice constant ~11.36 nm, combined with the capacitance calibrated by the Hall Effect, proves that the moiré feature is FFP. Secondly, the resistive peak at half fillings always coexists with a FFP peak. In the left (right) panel of Fig. 1d, there is only one moiré feature in the top and right (bottom and left) corner; the absence of another feature further away (where the carrier density is doubled) excludes the possibility of fractional fillings. Similar argument can be applied for the quarter filling, since usually correlated peaks are easier to form at half filling than at quarter filling. Overall, we can unambiguously identify the observed moiré feature as FFP.

1.2 The stacking angles

As crystallographic axes of graphene and hBN were not determined before stacking, we extracted these parameters of D1 from optical images and electrical measurement. In Fig. S1a, the misalignment between graphene and the top hBN flake is relatively larger (~2.3° or 32.3°) and that between graphene and the bottom hBN is almost negligible (~0° or 30°). Using the capacitance measured by the Hall Effect (Fig. S2b), we can determine the carrier density required to fully fill a moiré band (Fig. 1b and Fig. S2a) as n_s=3.475×10^{12} cm^{-2}. Hence the superlattice constant $\lambda = \sqrt{\sqrt{3}/8\, n_s}$=11.53 nm. The relation between λ and twisted angle θ reads[1]

$$\lambda = \frac{(1+\delta)a}{\sqrt{2(1+\delta)(1-\cos\theta)+\delta^2}} \quad (1)$$

where a=2.464 Å is the graphene lattice constant, δ=0.018 is the lattice difference between hBN and graphene. Finally, one obtains θ=0.69°. Compare this twisted angle with those derived from optical images, we prefer that it is the interface between graphene and bottom hBN that generates a moiré pattern. We note that the presented ferroelectricity in the main text is almost identical to the D2 device in this study (Supplementary Note 2) and H2, H5&H6 samples in previous reports[2]. Especially, for the D2 and H2 samples, the angles between two hBN flakes have been determined to be ~30° by SHG. Consequently,

the top hBN flake in our device is plausibly assumed to be 32.3° twisted with graphene.

1.2 Results of State 1

During several thermal cycles, D1 exhibits similar states but with various magnitudes of hysteresis. Although we have no clue how to manipulate them on purpose, these states could be switched from one to another. Actually, State 2 was firstly replaced by State 1 but eventually recovered.

Except the smaller hysteresis in Fig. S16a, we cannot find any qualitative difference between State 1 and State 2. In particular, we examine the dependence of anomalous screening on the displacement fields from the special (top) gate. As shown in Fig. S16b-c, we start or stop at V_t=0 V, but the anomalous screening and hysteretic behavior remain robust. It seems that only the turning of sweeping direction matters: whenever the CNP or FFP touches the boundary of a sweeping region in the forward (backward) direction, anomalous screening appears in the backward (forward) sweeping. More interestingly, if we shrink the sweeping range of V_t symmetrically, the sample may be stuck into the anomalous screening state (Fig. S17).

2. Measurement of Device 2 (D2)

2.1 The stacking angles

The crystallographic axes of graphene and hBN flakes were identified before stacking by Raman and SHG, respectively. As shown in Fig. S18, the top hBN is twisted by 2.7° with graphene, and the bottom hBN is 29.3°. The angle between two hBN flakes is around 32°.

2.2 Electrical measurement

A detailed characterization at the base temperature was performed in D2. Here the top gate is found to be the special gate and the bottom gate is the normal one. Similar to D1 in which the trivial gate is able to switch hysteresis loops, we find such a behavior in D2. As shown in Fig. S19a, a full hysteretic loop could be observed with scanning range ~0.2 V nm^{-1}. However, when shrinking the scanning range to 0.12 V nm^{-1}, the right half of hysteretic loops disappears owing to that the electronic states collapse into the stable anomalous states (Fig. S19b). It could be restored as shown in Fig. S19c, if the lower scanning limit is extended more than -0.2 V nm^{-1}.

Despite the similar performance of the trivial gate in D1 and D2, there are some subtle differences. In

D1, the larger scanning range leads to an unstable hysteresis and the states try to approach the stable states with anomalous screening. In opposite, for D2 a smaller range of the trivial gate (to be specific, on the negative side) turns out to be essential to eliminate the hysteresis (partially). More investigation is needed to explain the discrepancy on a quantitative level, which is extremely important to explore the switching functionality of a hysteretic device.

3. More control devices

To pin down the critical configuration for hysteretic behaviors, three types of devices were designed and prepared. Here we define the angle between graphene and the top (bottom) gate as θ_t (θ_b), and the angle between two hBN as Θ. As seen from Table S1, both top and bottom gates have ~0º alignment with the bilayer graphene in DG4 and DG6. However, neither hysteresis nor anomalous screening could be observed, despite the formation of a moiré superlattice. In contrast, when one of the gates is marginally twisted by 30º as in D2 and D1, hysteresis and anomalous screening immediately take place regardless of moiré features. It's important to stress that a device without moiré features does not mean the absence of moiré superlattices: the twisted angle that could be detected by electric measurement for our devices is less than ~2 degrees, due to the typical breakdown voltage of hBN flakes. For instances, in D2 (this study), the twist angle is around 2.7 degrees determined by optical measurement, but moiré features are in lack in resistance measurements. In such a device, a moiré superlattice is still present, although with a shorter superlattice constant and probably without electronic correlation.

In addition, to check whether the 30º twisted interface between graphene and hBN is the key factor, θ_b was set to be far away from symmetric axes while keeping θ_t~30º (DG8, 9 and 10). As expected, there was no moiré superlattice formed. Neither were hysteresis and anomalous screening. Overall, we can tentatively conclude that small-angle twisted interfaces and thus the resultant moiré superlattice are still essential to observe ferroelectricity in graphene heterostructures.

4. Discussion about the mechanism

Generally speaking, ferroelectricity could originate from pure electron dynamics or spontaneous lattice distortion. For the device studied in this work, there are only crystallographically aligned bilayer

graphene and hBN flakes, thus we discuss all possible electron- and/or lattice-driven mechanisms in terms of constituent layers and the interface.

a) Correlation induced interlayer charge transfer (within the bilayer graphene)

In this scenario, the essential object is the bilayer graphene, while the hBN flakes merely provide a special electrostatic environment. The bilayer graphene, subjected to perpendicular electric fields and a periodic superlattice potential, is endowed with layer-polarized moiré bands, *i.e.*, the electron and hole mini-bands reside in the bottom and top graphene, respectively. Electrons may exhibit strong correlation due to the narrow bandwidth of moiré bands. As shown in Fig. S20a, electrons firstly fill the valence band (the top layer in real space). Upon the band is half filled, the on-site energy U excludes double occupation within each moiré supercell, which is translated as band splitting in the momentum space. Compared with the energy gap Δ between conduction and valence bands, a larger U will result in prior population of the conduction band (the bottom layer in real space). In other words, electrons transfer from the top to the bottom layer, generating electric dipoles ordering between the two layers.

The above interlayer charge transfer model is able to qualitatively explain several key experimental observations, such as the difference between normal and special gates, the accompanied anomalous screening and so on. For the different gates, there may be only one hBN flake that forms a strong moiré pattern with graphene, whose corresponding min-band is narrow enough to introduce correlation for further Hubbard band splitting. Consequently, this gate behaves as a special gate and the other as a normal one. For the anomalous screening, during the process of band splitting, the gate voltage (of the special gate) needs to compensate the rising chemical potential, so that no additional carriers are introduced into the system, mimicking the ‘stop-working’ behavior. As a result, this scenario was previously believed to account for the moiré ferroelectricity, despite the lack of moiré features in experiments.

However, as pointed out in the manuscript, the basis of the above ICT model – correlated electrons – has not been scrutinized yet. Our devices with clear moiré features severely contradict with the moiré physics on a quantitative level. Firstly, the amount of transferred electrons is found to depend on specific status of the device (Fig. 3 in main text), which can far exceed the capacity of a moiré band. Such a quantitative comparison was in lack in literatures due to the missing moiré features. Secondly, the concomitant

anomalous screening can occur outside the moiré bands (on the electron and hole sides), *i.e.*, within dispersive bands (Fig. 2 in main text), which is against electronic correlation.

At last, we check the material dependence. Besides Bernal bilayer graphene, twisted bilayer graphene[4] and hBN intercalated bilayer graphene[3] also exhibit very similar ferroelectricity. The collection of these materials is actually very important to identify whether correlation is the key ingredient: While the former definitely has correlated electrons, the latter is expected to be free from electronic correlation. Therefore, graphene ferroelectricity seems to be independence of correlation, in line with our experimental results.

The new findings are in discrepancy with moiré bands of correlated electrons, challenging the ICT model. Nevertheless, it does not mean that an electron-driven mechanism is invalid; some new mechanisms that induce interlayer charge transfer is in demand

b) Sliding ferroelectricity (at the interface between graphene and hBN)

It is also possible that the ferroelectricity stems from lattice distortion, just like almost all conventional ferroelectric materials. Following the well-established sliding ferroelectricity, we plot a single graphene layer and the crystallographically aligned hBN in Fig. S20b. Note that the outer layer of graphene is omitted because it is much less influenced by the hBN owing to a large distance.

Inside a moiré supercell, there are two polar states at the graphene/hBN interface[5]: when half of the C atoms of the graphene layer locate right over the N atoms of hBN layer, the π electron cloud of the C atoms will be repelled by the N atoms with negative charge, giving rise to a vertical polarization downwards; in contrast, when half of the C atoms of the graphene layer locate right over the B atoms, the π electron cloud of the C atoms will be attracted and prolonged by the B atoms with positive charge, giving rise to a vertical polarization upwards. Note that when both B and N are overlaid by C atoms, the polarization is averaged out over the unit cell of graphene.

Although the two polar states could cancel each other over a moiré supercell, untwisted graphene/hBN interface may also exhibit ferroelectricity similar to the twisted bilayer hBN[6-8], where the macroscopic

polarization is switched by expanding/shrinking the area of polar states through domain wall motions. A theoretical justification is as follows:

The density functional theory (DFT) calculation was carried out using the projector augmented wave (PAW)[9] scheme with the Perdew–Burke–Ernzerhof (PBE) functional of generalized gradient approximation (GGA)[10] method as implemented in the Vienna *ab initio* simulation package (VASP)[11,12]. A plane wave cutoff of 750 eV was set in our calculations. K-point samplings of 12 × 12 × 1 was used. optB88 level[13] was used in our calculations to taking into consideration of the van der Waal forces. Atomic relaxation was performed until the force on each atom is smaller than 0.001 eV $Å^{-1}$, and the total energy change was less than 10^{-6} eV. The vacuum spacing is set as at least 16 Å along the out-of-plane direction that is sufficiently large. The electric field action is equivalent to exerting equal and opposite displacements on the oppositely charged atoms carrying Born effective charges in the unit cell[14], and in this work they are B and N atoms.

Two different stacking configurations of graphene and hBN are considered. The AB-stacking means the C atoms directly stacks on the B atoms and the N atoms stacks on the center of the hexagon in graphene. Similarly, the BA-stacking means the C atoms directly stacks on the N atoms and the B atoms stacks on the center of the hexagon in graphene. The crystal structures of AB-stacking and BA-stacking of graphene/hBN vdW heterostructure are depicted in Fig. S21a. We calculated the energy difference ΔU between AB-stacking and BA-stacking graphene/hBN vdW heterostructure under different electric field. As shown in Fig. S21b, it’s obvious that the energy of AB-stacking and BA-stacking is different under the same electric field. When the electric field is small, the energy of AB-stacking is always lower than that of BA-stacking. So the area of AB stacking (blue color in the right insets) is greatly enlarged, and the BA stacking (red color in the right insets) shrinks simultaneously. When the upward electric field continues to increase, ΔU changes from a negative value to a positive value, which means the energy of AB-stacking becomes higher than that of BA-stacking. Hence the proportion of AB and BA stacking areas is reversed.

As also shown in previous DFT calculations[5] for graphene/hBN heterobilayer, the vertical polarizations for the AB stacking configurations with C atoms right over B atoms and C atoms over N atoms are

respectively 1.5 pC/m and -0.33 pC/m, so the change in polarization after ferroelectric switching will be 1.83 pC/m, very close to that of MoS_2 bilayer (0.97 pC/m-(-0.97 pC/m)=1.94 pC/m). We note that the electrically tunable lattice relaxation and resulting polarization for MoS_2 bilayer have been simulated in a recent work[15], where the domains with opposite polarizations relax unevenly (one enlarged and the other one squeezed) under external electric field, giving rise to non-zero net polarization.

It's important to stress that *the above polarization is only valid in perpendicular electric fields*. Once the electric field goes to zero, the polarization will diminish accordingly, in contrast to the definition of ferroelectricity where a polarized states survives without the presence of an electrical field. Defects, mislocations or strain disorders may preserve the polarization. However, such extrinsic factors are uncontrollable and highly depend on local configurations of a specific device, which are not in line with the uniform and repeatable ferroelectricity in experiments.

Moreover, this lattice driven mechanism is difficult to explain the accompanied anomalous screening at present. There is another indirect evidence. Since the switching between two polar states is via domain wall motion, the two polar states of practical samples may coexist. The coexistence can be directly observed by microscopic characterization or – more easily – by electrical measurements. For example, in twisted bilayer hBN double resistance peaks always appear in the phase diagram[6], each of which represents either an upwards or downwards polar state. However, in our own samples and similar ones in the literature, such coexisting signals have never been observed. Instead, the polar states are verified to be quite uniform over the entire sample (Fig. S1c). All of these observations, although indirect, indicate that the graphene ferroelectricity is unlikely to be from a pure lattice distortion.

c) Sliding ferroelectricity (within hBN flakes)

The hBN flake may have stacking faults, invoking the sliding ferroelectricity naturally. However, this mechanism can be excluded completely. If such an imperfect hBN exists, both top and bottom hBN cut from the same thin crystal would enable hysteresis of resistivity, in direct contrast to the experimental results. Quantitatively, the polarized carrier in graphene is too large to be from sliding ferroelectricity in hBN because, as estimated in the manuscript, each layer of the hBN flake needs to be rhombohedrally stacked.

At last, we’d like to examine the origin of the special gate in this gate-specific ferroelectricity. Corresponding to the two possible scenarios (subsections a and b in the above), the requirement of a special gate is as follows: (1) In the ICT model, a possible explanation of distinct functionality is that the top and bottom hBN does not provide equally strong periodic modulation of electrostatic potential. The stronger one that controls the Hubbard band splitting, anomalous screening and ferroelectricity, would act as a special gate. (2) In the scenario of sliding ferroelectricity, the special gate must be associated with a polarized interface between hBN and graphene, e.g., two polar states may arise due to the inequivalent influence of B and N on the π electron cloud of C atoms.

To summarize, our results demonstrate that neither the electron-driven ICT model nor the lattice-driven sliding ferroelectricity can solely explain all the observation, hence the combination of the two may be required for a complete model.

Supplementary Tables

Table S1 Summary of device configurations and main results.

Device	Θ (°)	θ_t (°)	θ_b (°)	Moiré	Hysteresis	
					Top gate	Bottom gate
DG4	~0	~0	~0	yes	no	no
DG6	2.3	~1	3.3	yes	no	no
D2	32	2.7	29.3	no	yes	no
D1	31.3[a]	32[b]	0.69[b]	yes	yes	no
DG8	17.4	30.4	13	no	no	no
DG9	15	30	15	no	no	no
DG10	24	30	6	no	no	no

[a]This is determined by optical images combined with the device performance rather than a direct SHG measurement. See more details in Supplementary Note 1.2.

[b]Although the present angle assignment is preferred (Supplementary Note 1.2), we cannot unambiguously exclude the opposite case, *i.e.*, θ_b=0.69° and θ_t=32°.

<u>**Supplementary Figures**</u>

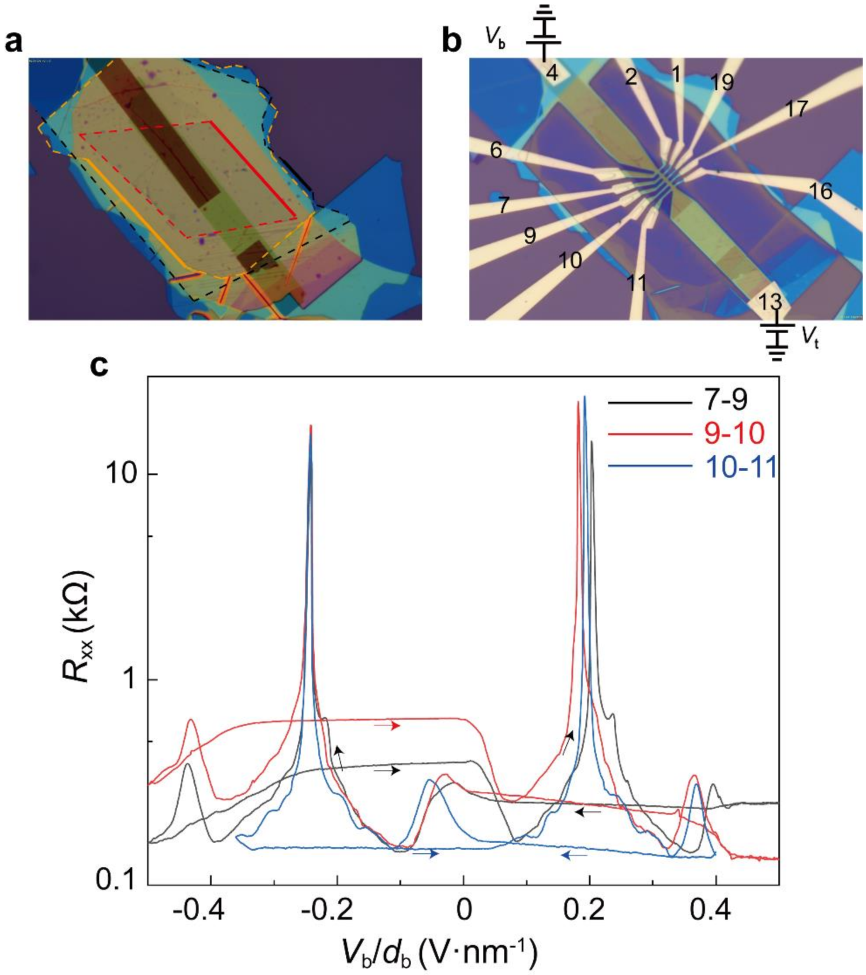

Fig. S1 Optical characterization of the ferroelectric sample D1. a. In the multilayer van der Waals heterostructure, a Bernal bilayer graphene (red) is roughly aligned with the top (black) and bottom (orange) hBN flakes. **b**. The finished device with indexed edge contacts. V_t and V_b denote the top and bottom gates, respectively. **c**. Uniform ferroelectric hysteresis over the entire sample. Three pairs of voltage probes show almost the same CNP and moiré features, although on the electron side there is a little shift.

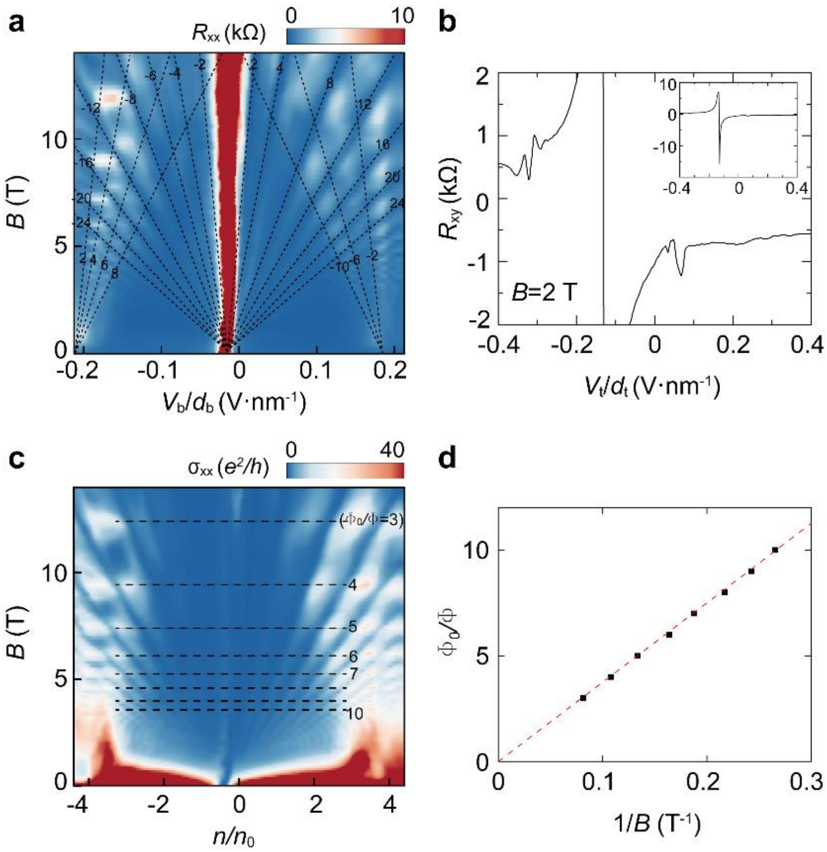

Fig. S2 Landau fan and Brown-Zak oscillations. a. From the Landau fan emanating from CNP, a bilayer graphene can be confirmed. Furthermore, second Dirac points are also unambiguously identified, whose Landau level sequences remain to be understood. **b**. The Hall Effect is conducted by sweeping V_t at 2 T, however, no sign reversal is observed at the second Dirac points, indicating the energy gap is not fully open. **c-d**. A superlattice constant ~11.36 nm can be derived from Brown-Zak oscillations, consistent with the one (~11.53 nm) determined directly from FFP.

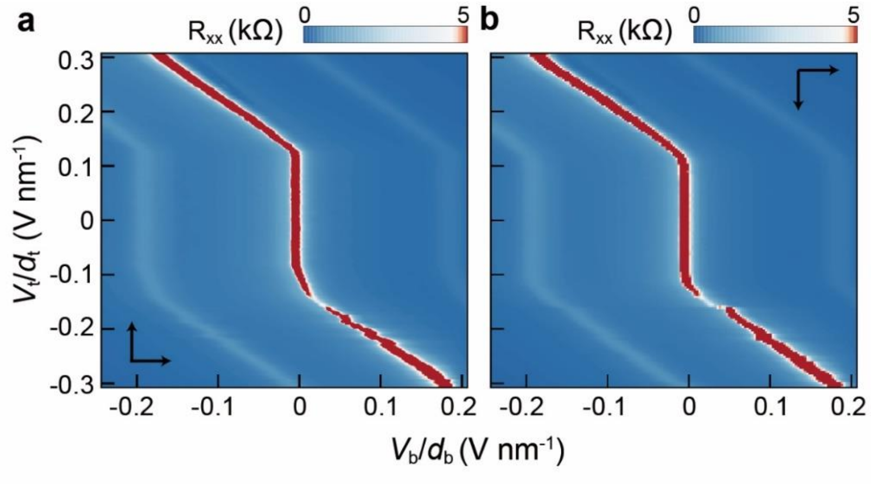

Fig. S3 Hysteresis loop disappears when the V_b scanning range exceeds the threshold of 0.15 V nm^{-1} and V_t scanning rate is extremely slow. Nevertheless, the anomalous screening remains robust. Here the fast-scanning axis is V_b, and the slow-scanning directions of V_t are upwards in **a** and downwards in **b**, respectively.

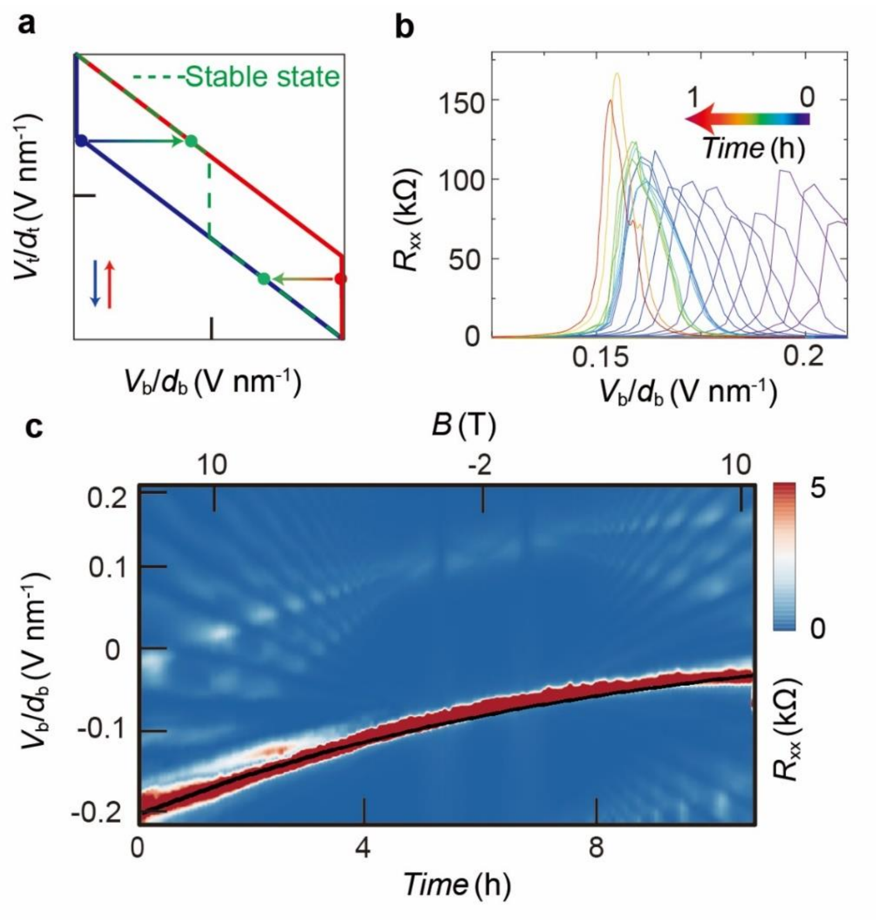

Fig. S4 Evaluation of relaxation time. a. In the schematic V_t-V_b phase diagram, the green dash curve represents the CNP position of stable states shown in Fig. 2b. The blue and red curves denote, were there no relaxation, CNP positions of downward and upward scanning of V_t, respectively. However, the actual case is that the CNP position will move gradually towards the stable state, which is highlighted by an arrow. As the relaxation time is found to be distinct for $V_t>0$ and $V_t<0$ V, two representative points are selected for detailed characterization. **b**. The time dependent transfer characteristics at the point of $V_t<0$ V in zero fields. **c**. The evolution at the point of $V_t>0$ V. Note the magnetic field varies from 14 to -2 T, then increases to 10 T again.

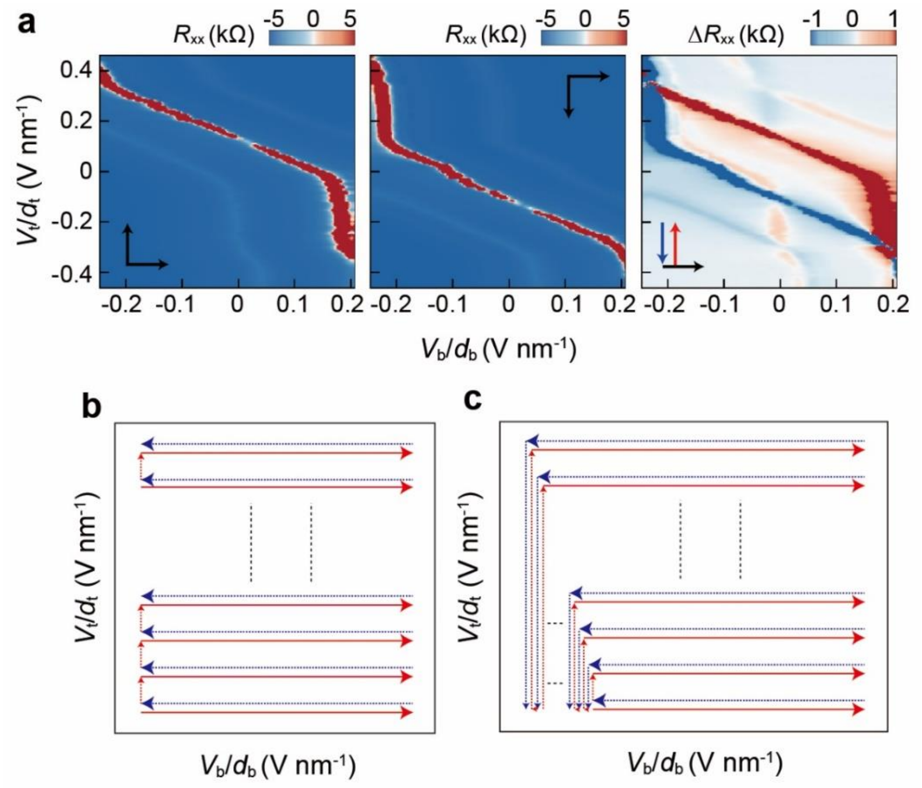

Fig. S5 Restoration of a full hysteresis for V_b/d_b>0.15 V nm^{-1}. a. When the scanning range of V_b is outside the relaxation-free regime, it is possible to obtain a full hysteretic loop by applying a special scanning scheme shown in **c**. **b-c**. Comparison of a normal (**b**) and the special scheme (**c**). In the latter, at the beginning of each scanning line (V_b) V_t is firstly reset to the limit and then scanned fast to the desired magnitude. In such a way, the time for relaxation is minimized to observed hysteresis.

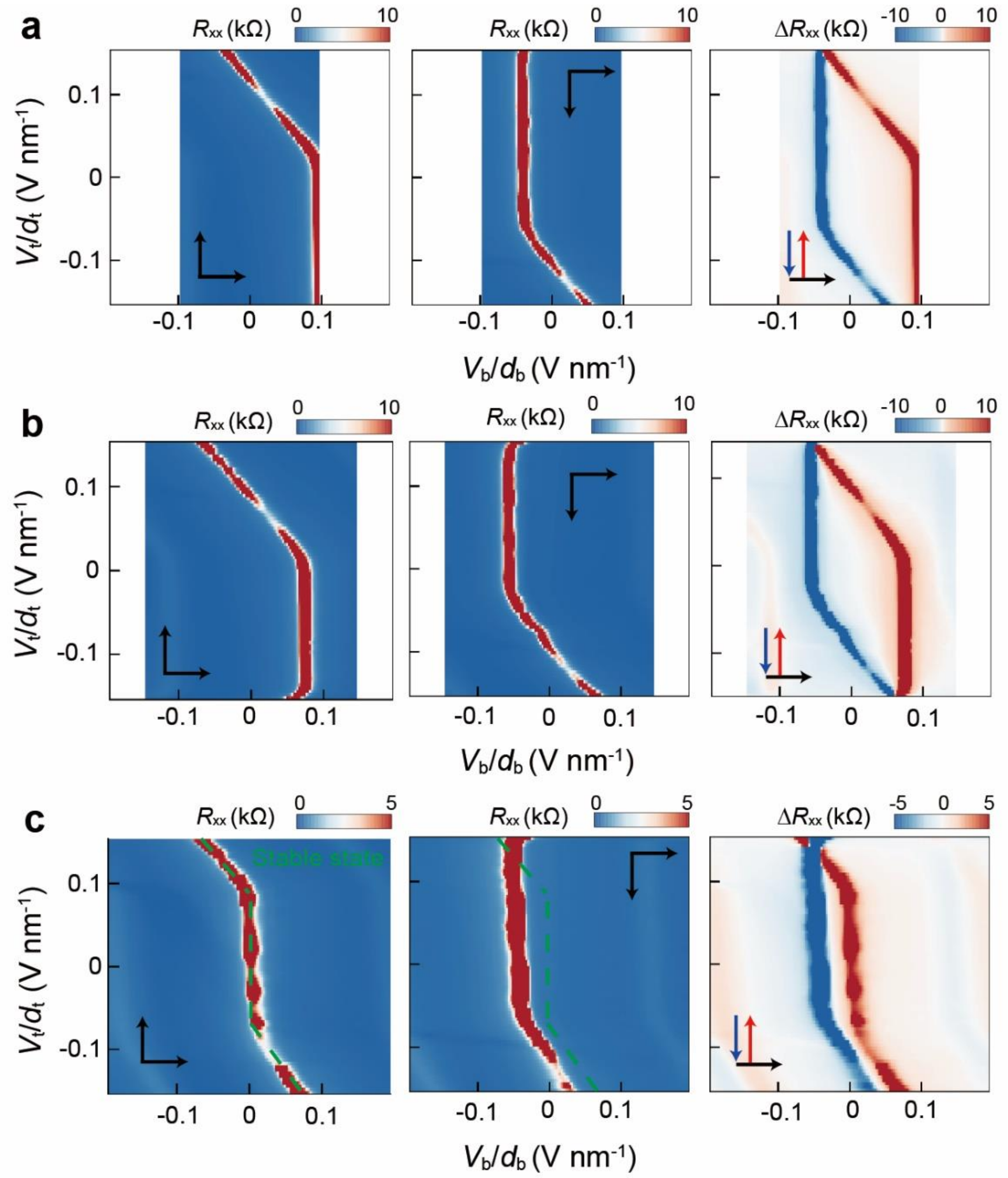

Fig. S6 Ferroelectric hysteresis depends on the scanning range of V_b. a. Raw data of the left panel in Fig. 2d. **b**. Raw data of the right panel in Fig. 2d. **c**. When the scan range expands to 0.2 V nm^{-1}, which is above 0.15 V nm^{-1}, the states are fully relaxed to the stable states for upward scanning but relatively weaker for the downward scanning. The resulting hysteresis loop is thus smaller than that in **a** and **b**.

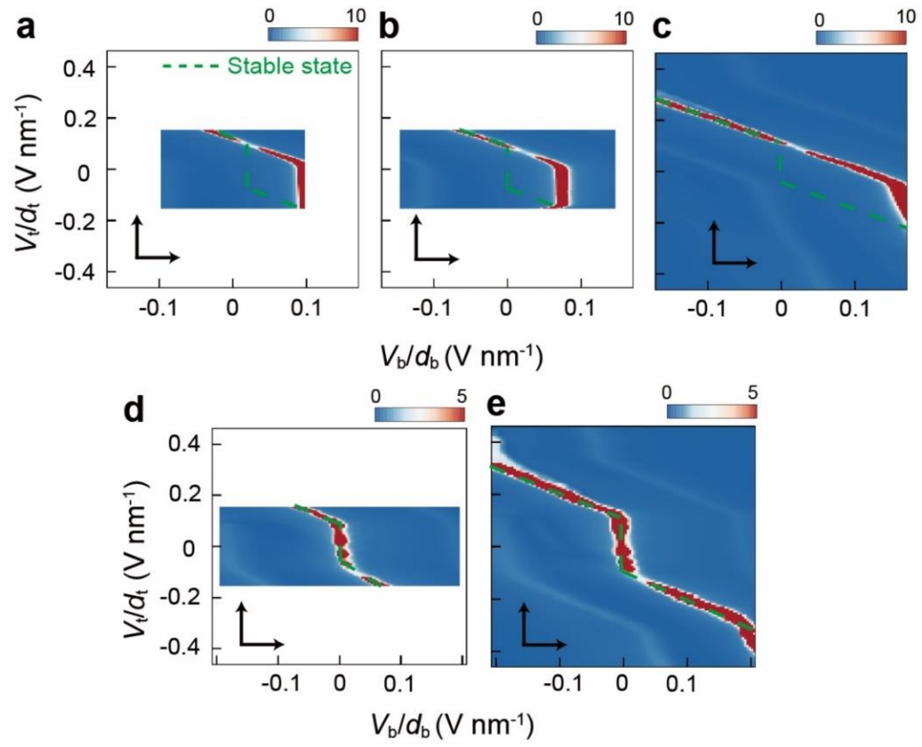

Fig. S7 Determining the threshold of relaxation in terms of V_b/d_b. Increasing the scanning range of V_b in a fine step, it is found that below 0.15 V nm^{-1}, a stable hysteresis could be identified (**a**, **b**), whereas the relaxation manifesting as the inclined CNP profile grows when above 0.15 V nm^{-1} (**c**). The states completely collapse into the stable states when approaching (**d**) and beyond (**e**) 0.2 V nm^{-1}.

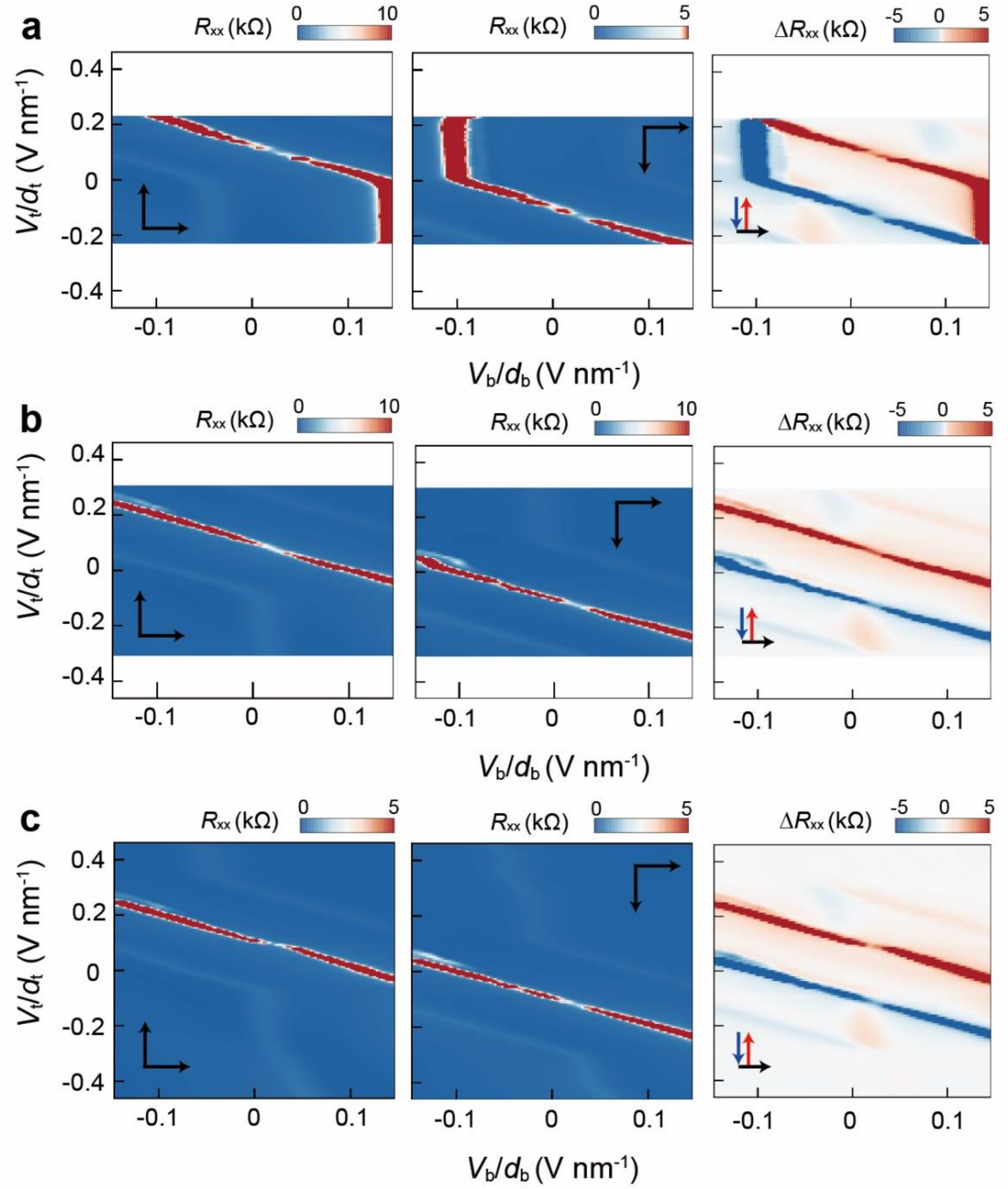

Fig. S8 Independence of ferroelectric hysteresis or relaxation on the scanning range of V_t. By shrinking the scanning range of V_t from 0.4 V nm^{-1}(**c**), the largest magnitude that is accessible without gate leakage, to 0.3 (**b**) and 0.2 V nm^{-1}(**a**), the stable hysteresis is repeatedly observed.

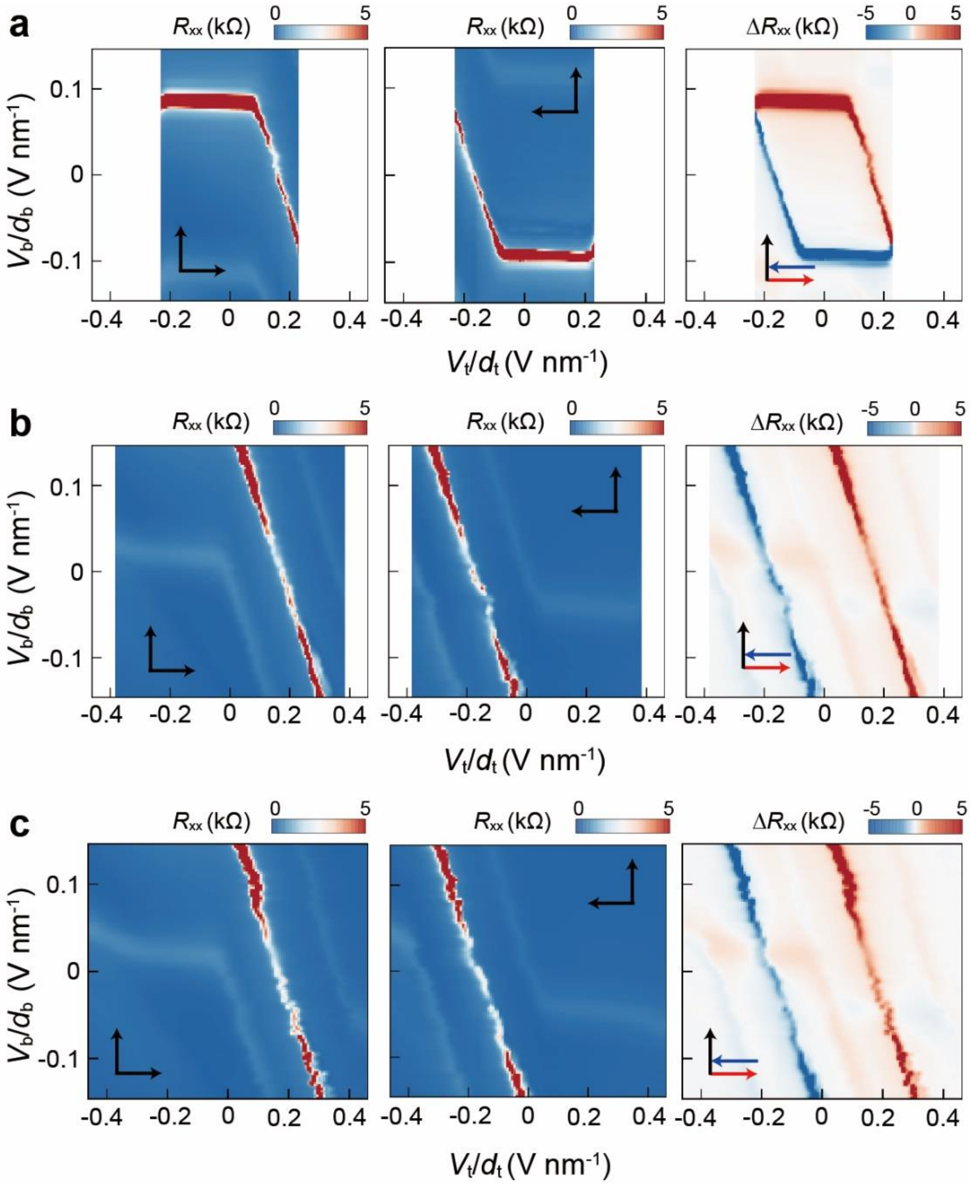

Fig. S9 Similar to Fig. S8, with the fast and slow scanning axes swapped. By shrinking the scanning range of V_t from 0.4 V nm^{-1}(**c**), the largest magnitude that is accessible without gate leakage, to 0.3 (**b**) and 0.2 V nm^{-1} (**a**), the stable hysteresis is repeatedly observed.

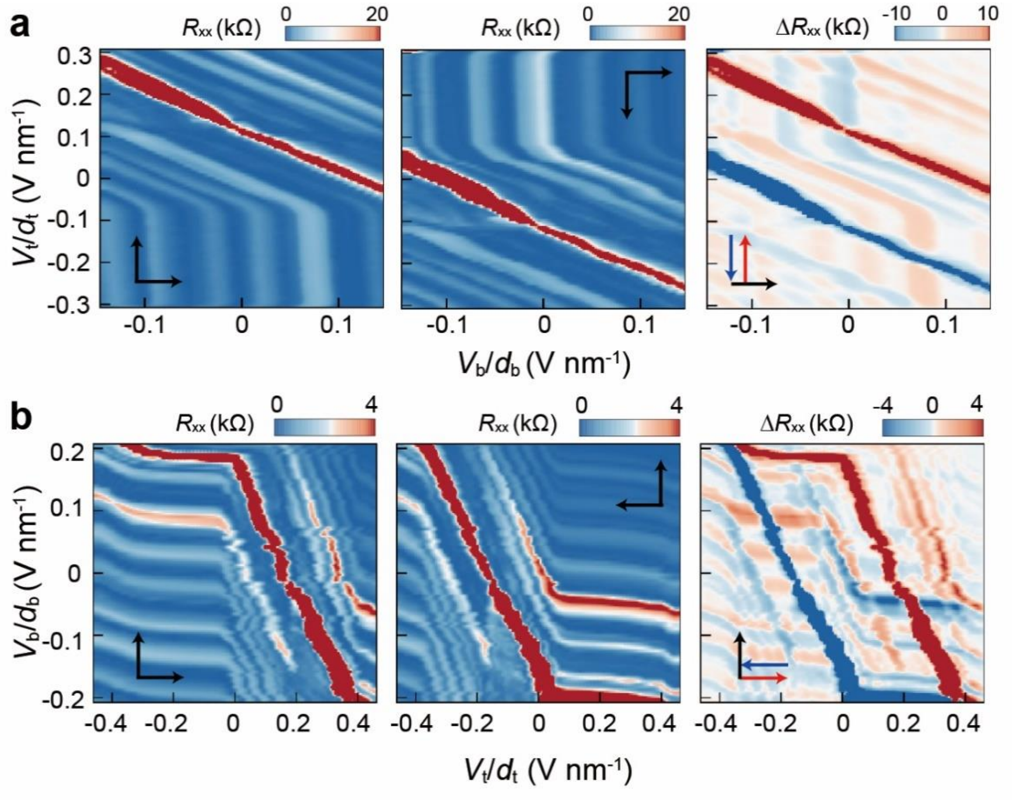

Fig. S10 Ferroelectricity in magnetic fields. a. Raw data for Fig. 3b, where the Landau levels form similar hysteretic loops as CNP and FFP in zero fields. **b**. Identical characterization with swapped fast and slow axes.

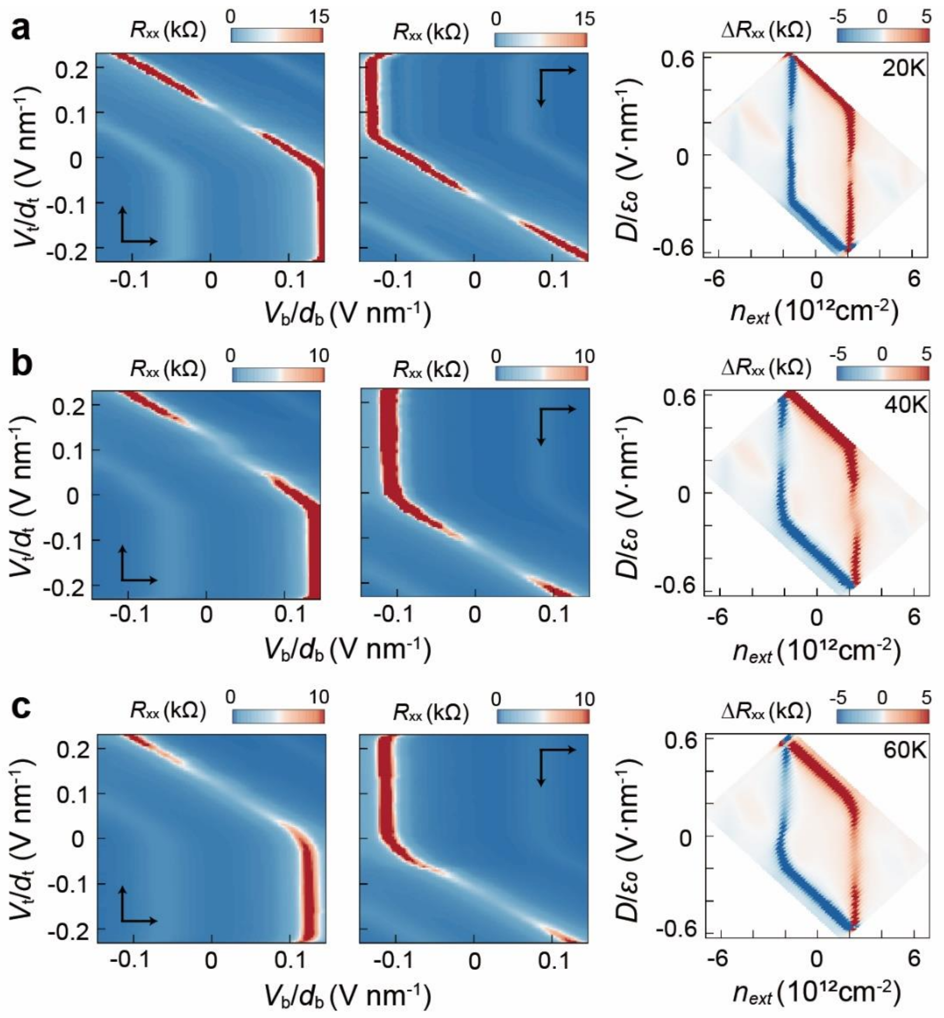

Fig. S11 Hysteretic loops are characterized at various temperatures. a. 20 K. **b**. 40 K. **c**. 60 K.

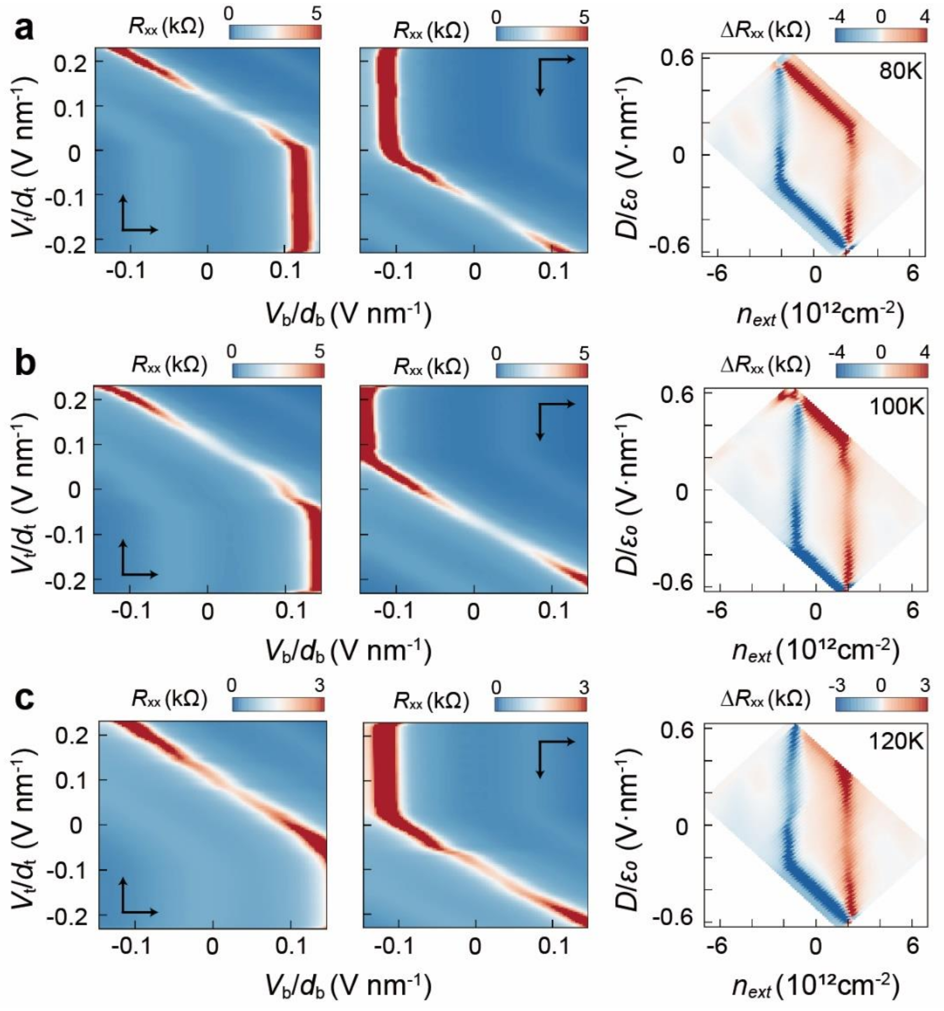

Fig. S12 Hysteretic loops are characterized at various temperatures. a. 80 K. **b**. 100 K. **c**. 120 K.

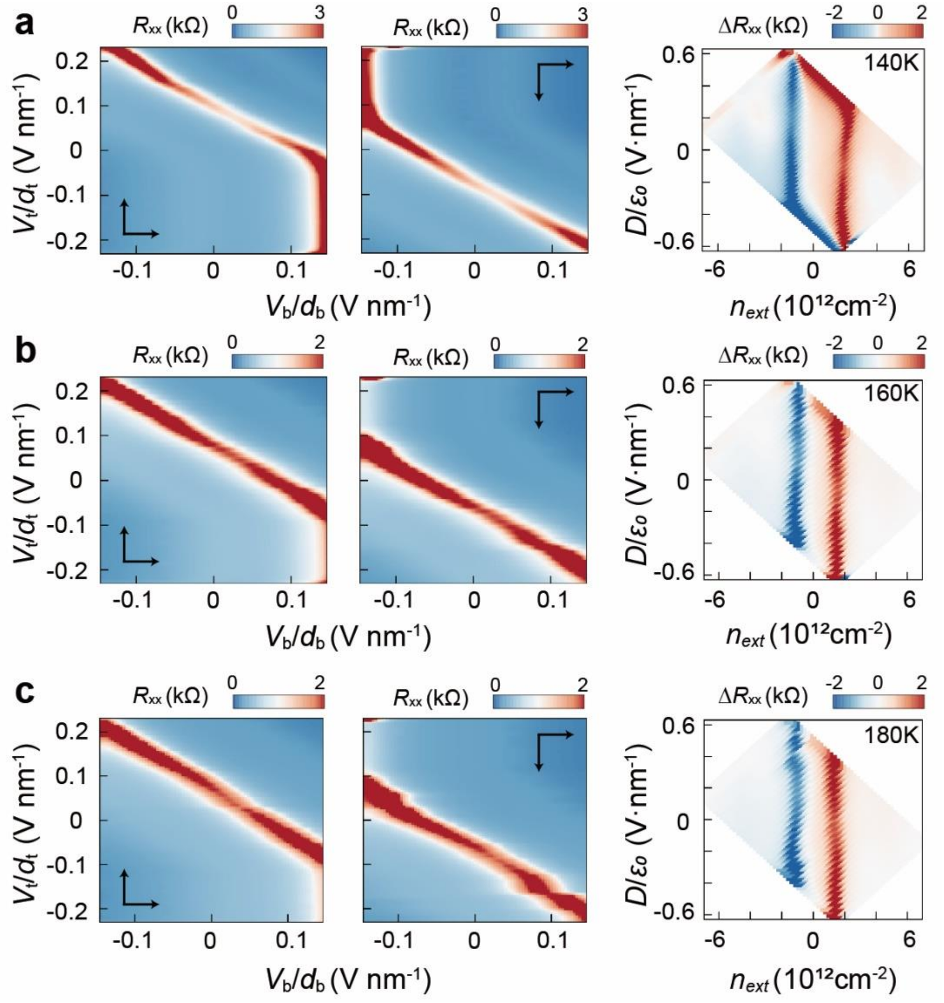

Fig. S13 Hysteretic loops are characterized at various temperatures. a. 140 K. **b**. 160 K. **c**. 180 K.

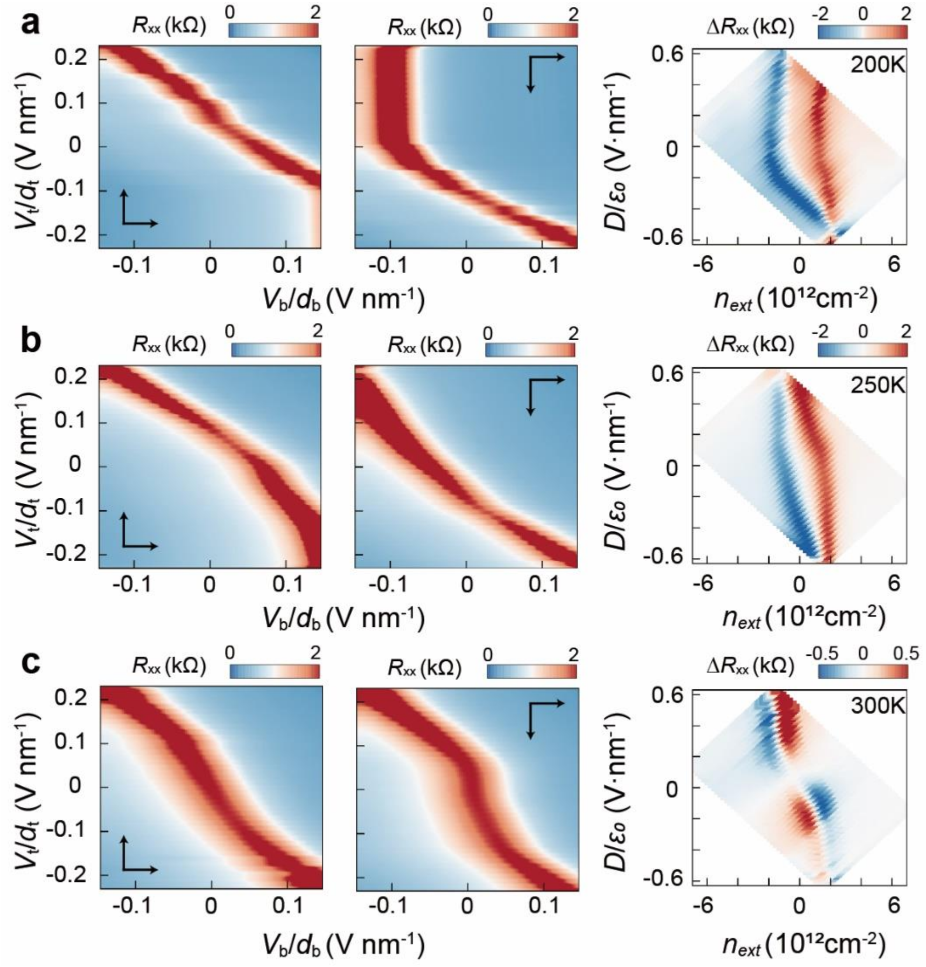

Fig. S14 Suppressed hysteresis at high temperatures. a. 200 K. **b**. 250 K. **c**. 300 K.

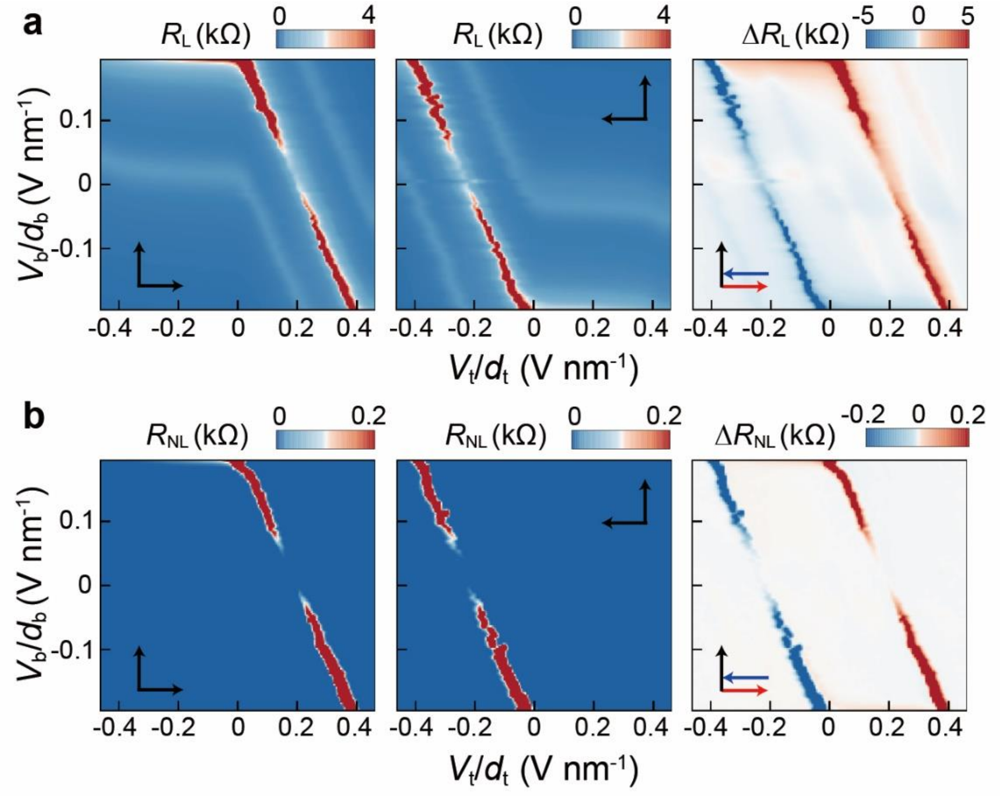

Fig. S15 Raw data for Fig. 4c-d. a. Local signal. **b**. Nonlocal signal.

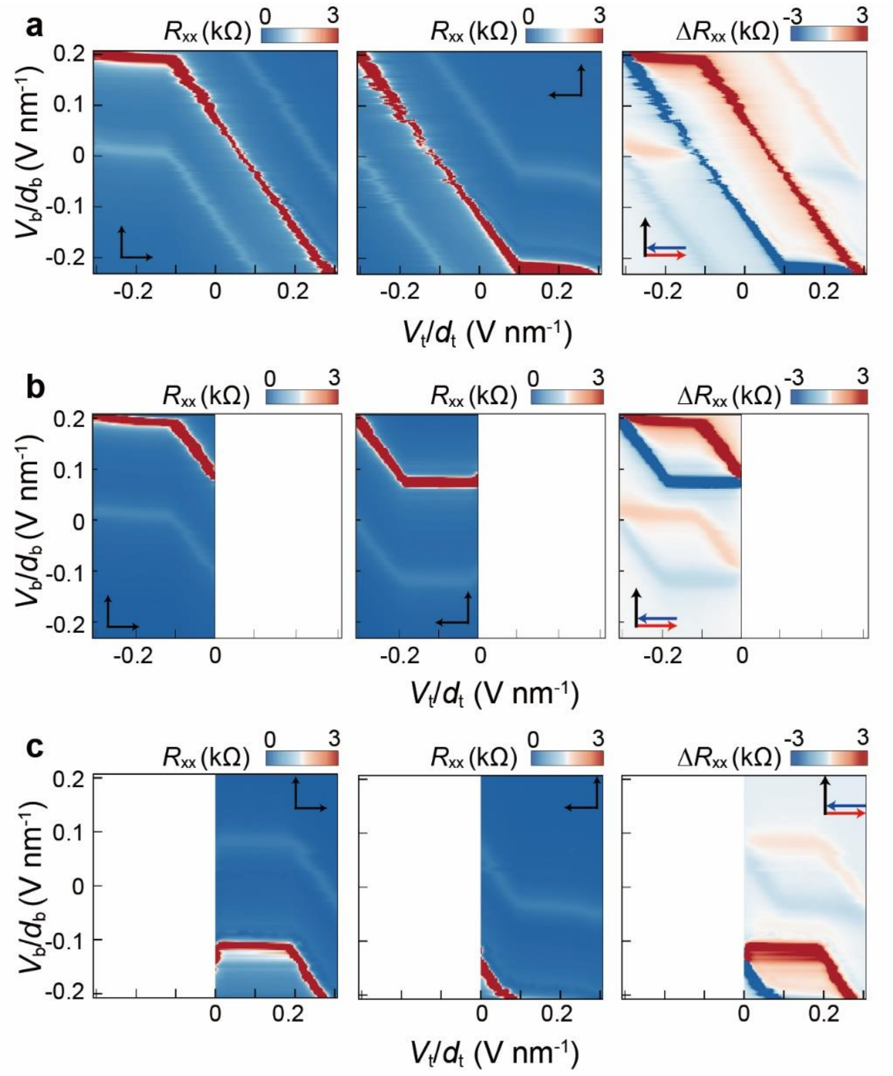

Fig. S16 Basic characterization of State 1. The ferroelectricity is robust, regardless the scanning range is symmetric (**a**), left half (**b**) or right half (**c**).

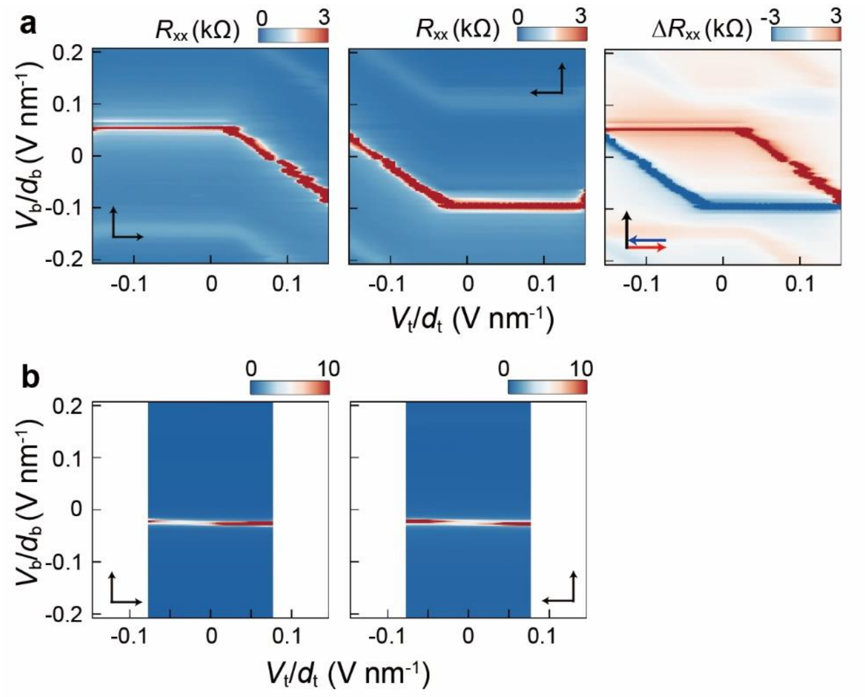

Fig. S17 Anomaly in the dependence of hysteretic loops on V_t. In State 1, normal ferroelectric hysteresis is observed when V_t/d_t is larger than the anomalous screening range (**a**), consistent with that in State 2. However, once it is smaller (**b**), the states cannot escape from the anomalous screening regime and hence the hysteresis disappears.

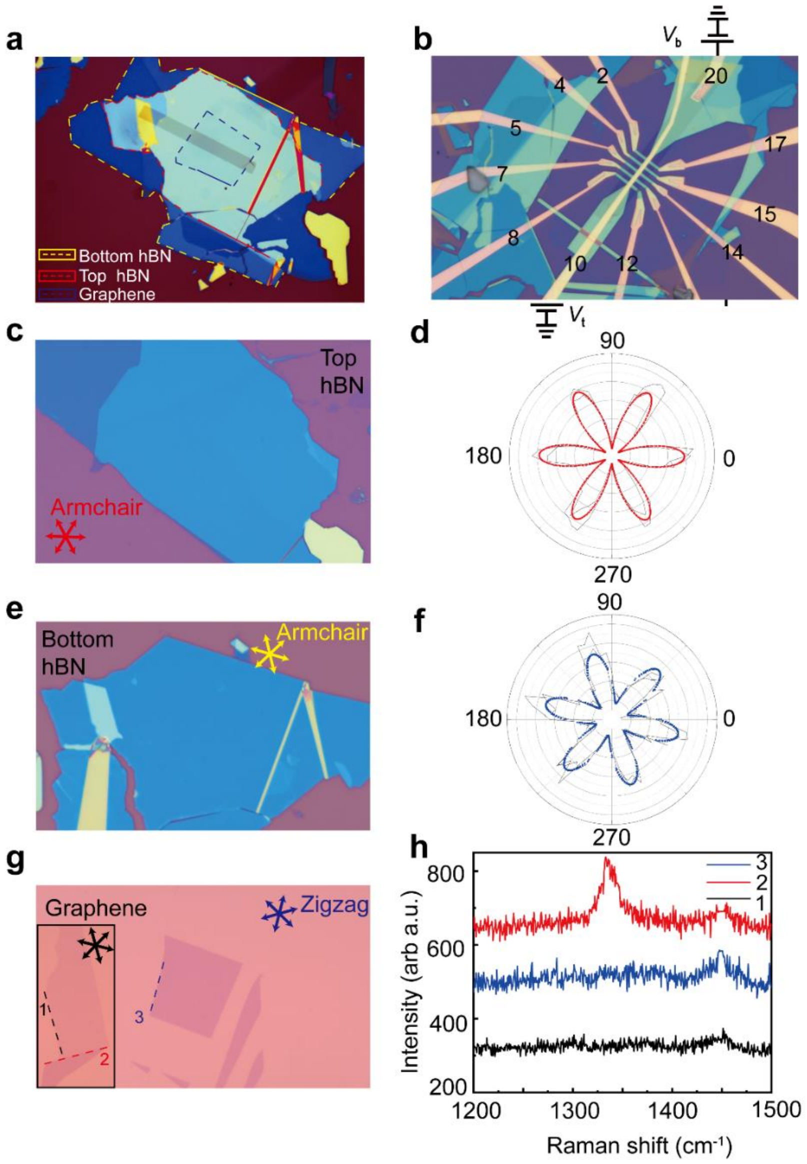

Fig. S18 Basic configurations of Device 2. a-b. Optical characterization of the van der Waals heterostructure before (**a**) and after fabrication of electrical contacts (**b**). The bottom, top hBN and the bilayer graphene are highlighted by yellow, red and blue dashed lines. **c-d**. Crystallographic axes determined by SHG for the top hBN. **e-f**. Crystallographic axes determined by SHG for the bottom hBN. **g-h**. Crystallographic axes determined by Raman for the bilayer graphene.

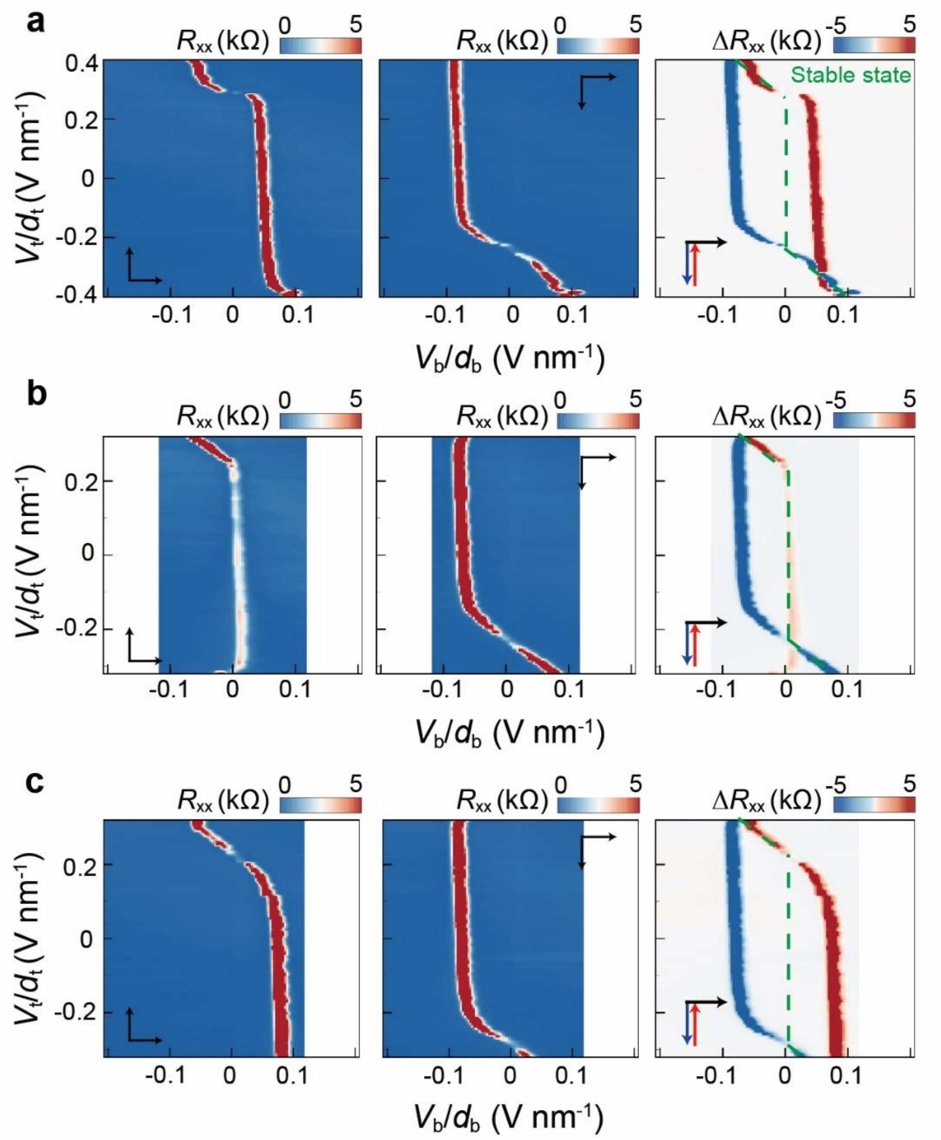

Fig. S19 Switching on/off the hysteresis by the trivial gate V_b in Device 2. a. When the trivial gate V_b/d_b is set as +/-0.2 V nm^{-1}, a full hysteretic loops could be observed. **b.** However, the right half of the loop is eliminated upon shrinking the scan range within ~0.12 V nm^{-1}. **c.** The full loop could be restored if extending the negative scan limit beyond -0.2 V nm^{-1}.

.

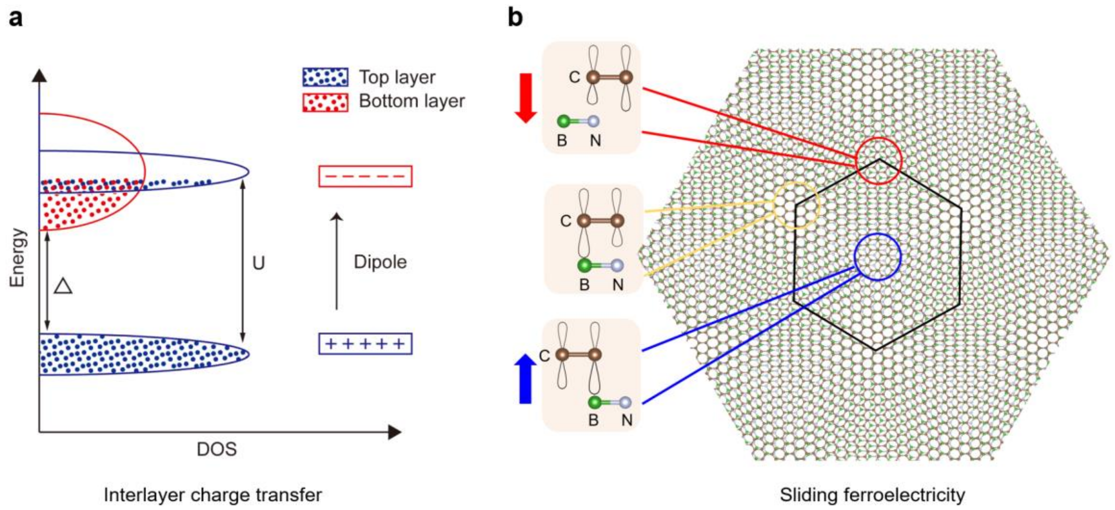

Fig. S20 Illustration of two types of ferroelectricity. **a.** Interlayer charge transfer model. The isolated moiré bands in the conduction (red) and valence (blue) bands are localized in the bottom and top layer in real space, respectively. The high-energy dispersive bands are omitted for clarity. Here the band gap Δ at charge neutral point is due to the perpendicular electric field, the correlation gap (on-site energy) U originates from the narrow bandwidth of a moiré band. The band splitting may induce electron transfer from one layer to another, forming spatially ordered electric dipoles. **b**. Sliding ferroelectricity at the interface of graphene and hBN flakes. When the twist angle is small or even zero degrees, relatively large domains (~ several nanometers) of polar states can be formed. Around the red (blue) circle where C atoms locate just above N (B) atoms, the π electron cloud of C atoms is repelled (elongated) to form a polar state. In contrast, in the yellow circle such polarization cancels out over a unit cell of graphene. With external electric field along perpendicular direction, the domain areas may be enlarged or squeezed, leading to a net polarization over a moiré supercell.

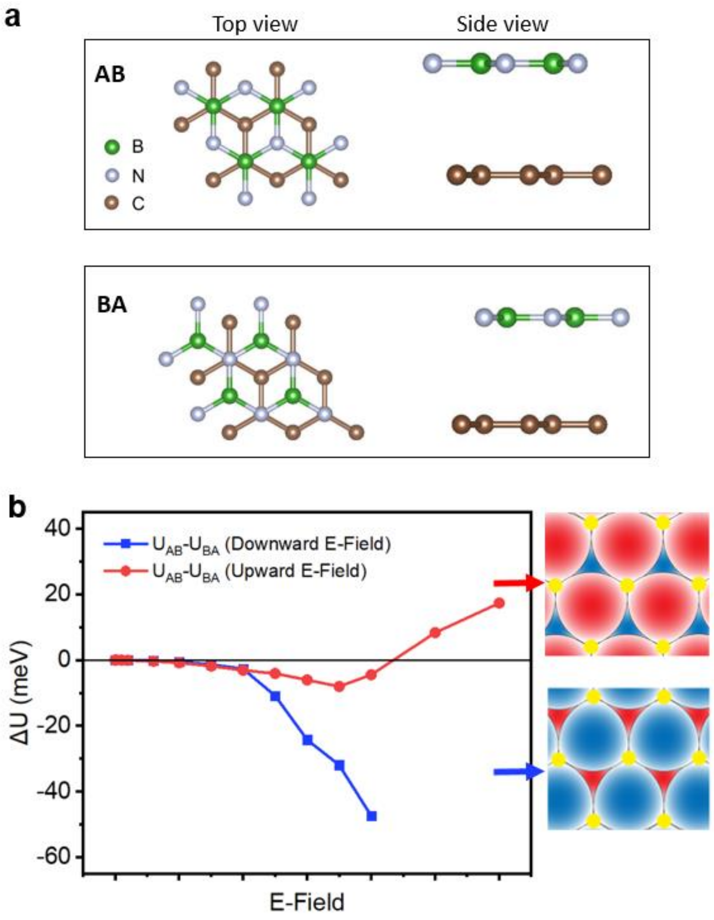

Fig. S21 DFT calculation of the polarization scheme in a moiré supercell shown in Fig. S20b. **a**. Atomic structures of AB- (top) and BA- (bottom) stacking of a graphene/hBN heterostructure with top and side views. **b**. The energy difference between AB- and BA-stacking under electric fields. The red line represents the downward electric field, and the blue line represents the upward electric field. Right insets: Schematics for the two polarized states in real space, where within a moiré supercell, blue (AB configuration) and red (BA configuration) regions are either enlarged or squeezed due to the positive/negative energy differences.

Supplementary References

1. Yankowitz, M. *et al.* Emergence of superlattice Dirac points in graphene on hexagonal boron nitride. *Nat. Phys.* **8**, 382–386 (2012).
2. Zheng, Z. *et al.* Unconventional ferroelectricity in moiré heterostructures. *Nature* **588**, 71–76 (2020).
3. Wang, Y. *et al.* Tunable ferroelectricity in hBN intercalated twisted double-layer graphene. *ArXiv210212398 Cond-Mat* (2021).
4. Klein, D. R. *et al.* Ferroelectric switching of superconductivity in magic-angle twisted bilayer graphene. *APS March Meeting* S60.010 (2022).
5. Li, L. & Wu, M. Binary Compound bilayer and multilayer with vertical polarizations: Two-dimensional ferroelectrics, multiferroics, and nanogenerators. *ACS Nano* **11**, 6382–6388 (2017).
6. Yasuda, K., Wang, X., Watanabe, K., Taniguchi, T. & Jarillo-Herrero, P. Stacking-engineered ferroelectricity in bilayer boron nitride. *Science* **372**, 1458–1462 (2021).
7. Vizner Stern, M. *et al.* Interfacial ferroelectricity by van der Waals sliding. *Science* **372**, 1462–1466 (2021).
8. Woods, C. R. *et al.* Charge-polarized interfacial superlattices in marginally twisted hexagonal boron nitride. *Nat. Commun.* **12**, 347 (2021).
9. Blöchl, P. E., Projector augmented-wave method. *Phys. Rev. B.* **50**, 17953–17979 (1994).
10. Perdew, J. P., Burke, K., Ernzerhof, M., Generalized gradient approximation made simple. *Phys. Rev. Lett.* **77**, 3865–3868 (1996).
11. Kresse, G., Furthmüller, J., Efficient iterative schemes for ab initio total-energy calculations using a plane-wave basis set. *Phys. Rev. B.* **54**, 11169–11186 (1996).
12. Kresse, G., Furthmüller, J., Efficiency of ab-initio total energy calculations for metals and semiconductors using a plane-wave basis set. *Comp. Mater. Sci.* **6**, 15–50 (1996).
13. Klimeš, J., Bowler, D. R., and Michaelides, A., Chemical accuracy for the van der Waals density functional. *J. Phys.: Cond. Matter* **22**, 022201 (2010).
14. Liu, J., Liu, S., Yang, J., Liu, L., Electric, Auxetic Effect in Piezoelectrics. *Phys. Rev. Lett.* **125**, 197601 (2020).
15. Bennett, D., Remez, B. On electrically tunable stacking domains and ferroelectricity in moiré superlattices. *npj 2D Mater. Appl.* **6**, 7 (2022).
